# Inhibition of Vascular Smooth Muscle Cell Proliferation by *Gentiana lutea* Root Extracts

**DOI:** 10.1371/journal.pone.0061393

**Published:** 2013-04-18

**Authors:** Rushendhiran Kesavan, Uma Rani Potunuru, Branislav Nastasijević, Avaneesh T, Gordana Joksić, Madhulika Dixit

**Affiliations:** 1 Laboratory of Vascular Biology, Department of Biotechnology, Indian Institute of Technology Madras, Chennai, India; 2 Department of Physical Chemistry, Vinca Institute of Nuclear Sciences, Belgrade, Serbia; University of Nebraska Medical Center, United States of America

## Abstract

*Gentiana lutea* belonging to the *Gentianaceae* family of flowering plants are routinely used in traditional Serbian medicine for their beneficial gastro-intestinal and anti-inflammatory properties. The aim of the study was to determine whether aqueous root extracts of *Gentiana lutea* consisting of gentiopicroside, gentisin, bellidifolin-8-O-glucoside, demethylbellidifolin-8-O-glucoside, isovitexin, swertiamarin and amarogentin prevents proliferation of aortic smooth muscle cells in response to PDGF-BB. Cell proliferation and cell cycle analysis were performed based on alamar blue assay and propidium iodide labeling respectively. In primary cultures of rat aortic smooth muscle cells (RASMCs), PDGF-BB (20 ng/ml) induced a two-fold increase in cell proliferation which was significantly blocked by the root extract (1 mg/ml). The root extract also prevented the S-phase entry of synchronized cells in response to PDGF. Furthermore, PDGF-BB induced ERK1/2 activation and consequent increase in cellular nitric oxide (NO) levels were also blocked by the extract. These effects of extract were due to blockade of PDGF-BB induced expression of iNOS, cyclin D1 and proliferating cell nuclear antigen (PCNA). Docking analysis of the extract components on MEK1, the upstream ERK1/2 activating kinase using AutoDock4, indicated a likely binding of isovitexin to the inhibitor binding site of MEK1. Experiments performed with purified isovitexin demonstrated that it successfully blocks PDGF-induced ERK1/2 activation and proliferation of RASMCs in cell culture. Thus, *Gentiana lutea* can provide novel candidates for prevention and treatment of atherosclerosis.

## Introduction

Pathogenesis of atherosclerosis and neo-intimal thickening post angioplasty involves excessive migration and proliferation of smooth muscle cells (SMCs) from media into the lumen of blood vessels. Increased expression of several growth factors such as basic fibroblast growth factor (bFGF) and platelet-derived growth factor (PDGF)-BB contribute to atheroma formation [1]. These agonists by activating the PI-3kinase and/or the mitogen activated protein kinase (MAPK) pathways promote migration and proliferation of vascular smooth muscle cells leading to their subsequent accumulation in the plaque [2], [3].

Plant derived extracts have been extensively used in traditional medicine worldwide. These phytochemicals, which are rich in flavonoids and xanthones, exhibit beneficial health effects by imparting protection against hepato- or neuro-toxicity [4]. For example, members of the Gentianaecae family are used in Serbian and Peruvian folk medicines to treat digestive disorders. Similarly, Swertia plants also belonging to the Gentianaecae family are in use in Asian medicine for their anti-inflammatory properties [5], [6]. Additionally, xanthones and glucosides obtained from *G lutea*, and *G austriaca* extracts have radio-protective properties [7]–[9]. Active principles of these extracts include the bitter tasting secoiridoids such as gentiopicroside, amarogentin and swertiamarin, and flavonoids and xanthones such as isovitexin and isogentisin respectively [10], [11]. Analysis of several commercially available *G lutea* extracts have demonstrated that the most abundant compound in them is gentiopicrioside (4.46–9.53%) followed by swertiamarin and xanthone glycosides, while gentisin and isogentisin are seen in much lower concentration (0.02–0.11%) [10]. Work done with purified isogentisin, has demonstrated that it protects endothelial cells from cigarette smoke induced cell death [12] while gentiopicriocide exhibits smooth muscle relaxing effects [13]. These studies hence suggest that Gentiana species may have beneficial cardio-vascular effects however the molecular mechanisms employed by these compounds are currently ill-understood. In this study, we investigated the role of *G lutea* root extract on PDGF-BB induced proliferation of primary cultures of rat aortic smooth muscle cells (RASMCs). Additionally we examined the effects of *G lutea* extract on PDGF-BB induced cell cycle progression and signal transduction involving ERK1/2 activation and iNOS expression.

## Materials and Methods

### 2.1 Materials Used

DMEM-F12 medium and antibiotic solution consisting of penicillin and streptomycin were from HiMedia Labs, Mumbai, India. Fetal bovine serum of South American origin was from GIBCO, Invitrogen, NY, USA. Antibodies against phospho- and total forms of eNOS, ERK1/2, PDGFR-β, IKKα, iNOS, cyclinD1 and PCNA were from Cell Signaling Technology Inc., Beverly MA, USA. EKR1/2 activation inhibitor (328000) was from Calbiochem, Inc. La Jolla, USA. PDGF-BB, EGF, bFGF, Insulin, Elastase, Collagenase, Trypsin inhibitor, iNOS PCR primers, L-arginine, Diaminofluorescein –2 Diacetate (DAF2-DA), Alamar blue reagent, propidium iodide, NOS inhibitor L-NAME and all the other dry lab chemicals were from Sigma Aldrich, St. Louis, MO, USA. HPLC grade acetonitrile and methanol were from J.T.Baker (Deventer, Netherlands).

### 2.2 Plant Material and Extract Preparation

*Gentiana lutea* plant roots were purchased from the Institute of Medicinal Plant Research “Dr. Josif Pancic”, Belgrade, Serbia. Aqueous root extracts were prepared by boiling the gentian roots in water (in ratio 1∶20, m/V) for 10 minutes, followed by filtration though 0.45 µm filters (Millipore Co. Ltd) as described previously [14], [15]. Frozen extracts were lyophilized and stored in sample tubes until further analysis at a dry place. At the time of experimentation the extracts were reconstituted in sterile distilled water (at a concentration of 10 mg/ml) and filtered through 0.22 µm filter for further use.

### 2.3 Isolation and Culture of Smooth Muscle Cells

Male Wistar rats weighing 100–150 g were procured from Kings Institute, Chennai. The protocol employed for isolation and culture of aortic smooth muscle cells was approved by the Institutional Animal Ethics Committee at the Indian Institute of Technology Madras in accordance with Indian Council of Medical Research, Government of India guidelines. These guidelines are formulated in accordance with U.S. National Institutes of Health guidelines for care and use of laboratory animals. Thoracic aorta were obtained and subjected to collagenase based enzymatic digestion for isolation of smooth muscle cells as previously published [16]. Cells were seeded and cultured on to collagen coated 6-well tissue culture dishes in DMEM-F12 medium supplemented with 10% (v/v) FBS, penicillin, streptomycin, fibroblast growth factor and epidermal growth factor in a humidified atmosphere of 5%CO_2_–95% air as reported previously [16]. Prior to experimental treatments, cells were serum-starved over-night. All experiments were done with quiescent cells up to passage two.

### 2.4 Immunoblotting

Cells were washed with ice-cold phosphate buffer saline followed by lysis in Triton X lysis buffer (20 mmole/LTris, 100 mmole/L NaCl, 10% triton X-100, 1 mmoleL/L EDTA, 1 mmole/L sodium orthovanadate, 2.5 mmole/L sodium pyrophosphate, 0.5% sodium deoxycholate, 1X protease inhibitor from Sigma). Following separation on polyacrylamide gels, proteins were electrophoretically transferred to polyvinylidenedifluoride (PVDF) membranes from Amersham. Transferred proteins were incubated with respective primary and secondary antibodies as per manufacturer’s instructions and equal loading of blots was confirmed by re-probing the blots with β-actin. Enhanced chemiluminescence based ECL plus detection kits were obtained from Cell Signaling Technology Inc. Beverly, MA, USA.

### 2.5 Nitric Oxide (NO) Measurements via DAF2-DA Imaging

Cultured SMCs were serum starved prior to treatment with PDGF-BB (20 ng/ml) in presence or absence of *Gentiana lutea* extract for given time duration. For measurement of intracellular NO, cells were co-incubated with 10 µmole/L DAF2-DA and 1 mmole/L L-arginine as nitric oxide synthase substrate. Release of NO was scored as appearance of green fluorescence due to binding of cellular NO to DAF2 dye. DAF2-DA is a cell permeable dye, which loses its diacetate moiety upon entering the cell due to action of cellular esterases and fluoresces upon binding to NO [17]. Images were captured using Olympus immunofluoroscence microscope equipped with ProgResCapturePro 2.7 camera followed by analysis with ImageJ software (NIH).

### 2.6 Alamar Blue Assay

Cell viability was measured by Alamar Blue assay [18]. Briefly, Alamar blue (Resazurin sodium salt from Sigma) was dissolved in phosphate buffered saline pH 7.4 to make a stock of 1 mg/mL and a final working concentration of 0.1 mg/mL in cell culture medium. Resazurin is a redox indicator, which measures the reducing environment of the cell by reducing to a pink colored resorufin. Following experimental treatments with PDGF-BB (20 ng/ml, 24 hours) in presence or absence of Gentiana extract (1 mg/ml), the cells were treated with alamar blue dye. Colour change was monitored colorimetrically at 590 nm and 570 nm to evaluate oxidized versus reduced forms respectively of the reagent by using multi-mode plate reader from Spectramax.

### 2.7 Cell Cycle Analysis

RASMCs were seeded on collagen coated 6-well dishes and were maintained in growth medium until they reached 70% confluence. They were then serum starved over-night for synchronization followed by treatment with PDGF-BB (20 ng/ml) with or without *G lutea* extract for up to 24 hours. Cells were trypsinized, centrifuged at 12000 rpm for 10 minutes and the pellets were re-suspended in 0.3 ml PBS. Cell fixation was done with ice-cold 70% ethanol at 4°C for 16 hours. Fixed cells were vortexed and briefly centrifuged at 12000 rpm for 5 minutes, followed by re-suspension of pellet in PBS containing 10 mg/ml RNAaseA and propidium iodide (50 µg/ml) for staining. Following incubation at 37°C for 1 hour, the PI-DNA complex in the nucleus of each cell was measured using FACS Diva (Becton and Dickinson Co., Franklin Lakes, NJ, USA). Subsequent analysis to determine percentage of cells in various stages of cell cycle was done using FlowJo software.

### 2.8 Docking Analysis

Docking analysis of *Gentiana lutea* constituents onto human MEK1 were performed using the Lamarckian genetic algorithm of AutoDock4 [19]. Putative docking sites were first identified using the entire macromolecules as search space followed by rigid docking at the sites of high binding energy. The PDB ids of MEK1 used for the analysis were 3EQF and 3EQH [20]. These are crystal structures of ternary complexes of MEK1 with Mg^2+^ATP and inhibitors K252a and U1026 respectively. The chemical ids of the ligands used for the study were: CID 9912413 for K252A, CID 3006531 for U1026, CID 88708 for gentipicroside, CID 115149 for amarogentin, CID 162350 for isovitexin, CID 442435 for swertiamarin, and CID 5281636 for gentisin. The search parameters for docking analysis were as follows: Grid parameters: number of points, 62 × 62 × 62, spacing: 0.375 Å (Rigid docking), 1.0 Å (Blind docking), number of runs: 100, initial population size: 150, maximum number of evaluations: 2500000, maximum number of generations: 27000 and default values were used for all the other parameters.

### 2.9 Statistical Analysis

Results are expressed as mean±SEM for a minimum of four independent experiments and statistics were performed using Student’s t-test using GraphPad Prism software. P values <0.05 were considered to be statistically significant.

## Results

### 3.1 Determination of Cytotoxic Concentrations of the Extract

**Figure 1:**
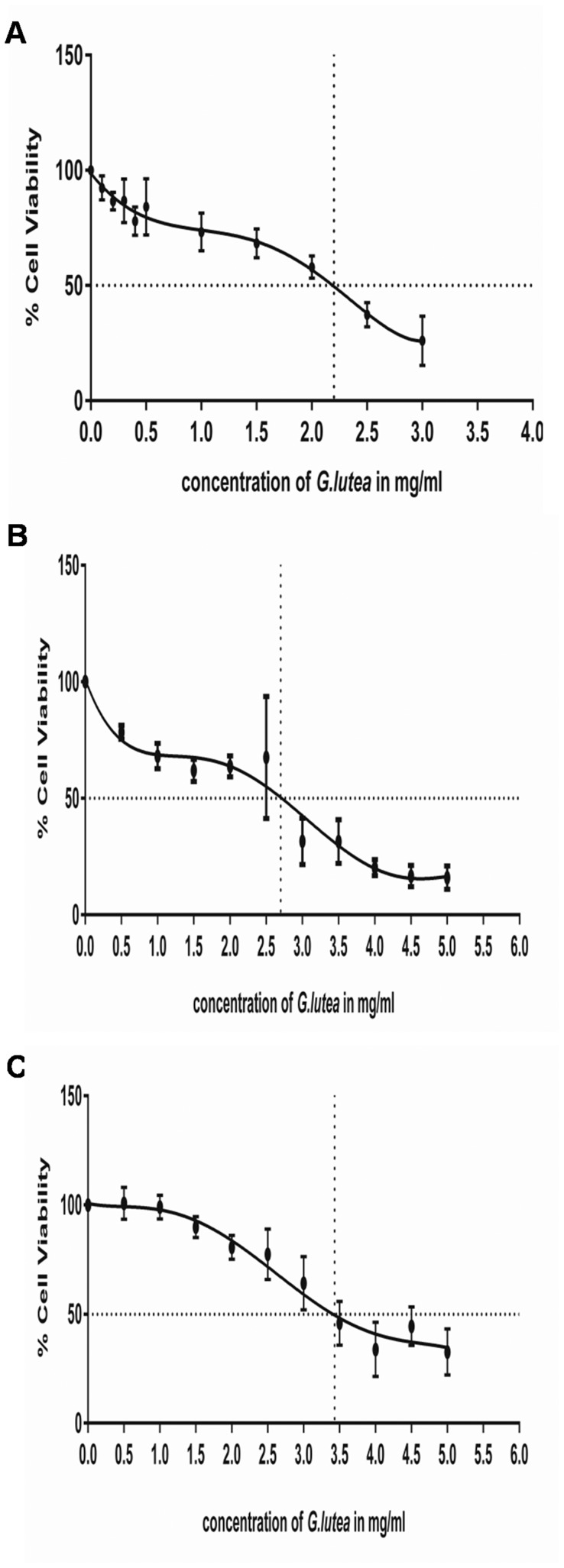
IC_50_ values for the *G. lutea* extract (1 mg/ml) on smooth muscle cells. A) Primary cultures of rat aortic smooth muscle cells (RASMCs), B) Rat aortic smooth muscle cell line A7r5 and C) Human aortic smooth muscle cell line ATCC-CRL-1999.

UPLC and MALDI-TOF analyses previously carried out on the extract [14], reported presence of following compounds: gentisin, bellidifolin-8-O-glucoside, demethylbellidifolin-8-O-glucoside, isovitexin, swertiamarin, amarogentin and getiopicroside. Among these, the major constituent of the extract was gentiopicroside as already reported [14]. We first determined the concentration of the extract at which it will inhibit proliferation by 50% (IC_50_) for primary cultures RASMCs, rat and human specific aortic smooth muscle cell lines A7r5 and ATCC CRL-1999 respectively. These were found to be 2.22 mg/ml for primary cultures of RASMCs, 2.7 mg/ml for A7r5 and 3.43 mg/ml for human cell line as shown in figure 1.

### 3.2 Effect of Extract on PDGF-BB Induced Cell Proliferation

**Figure 2:**
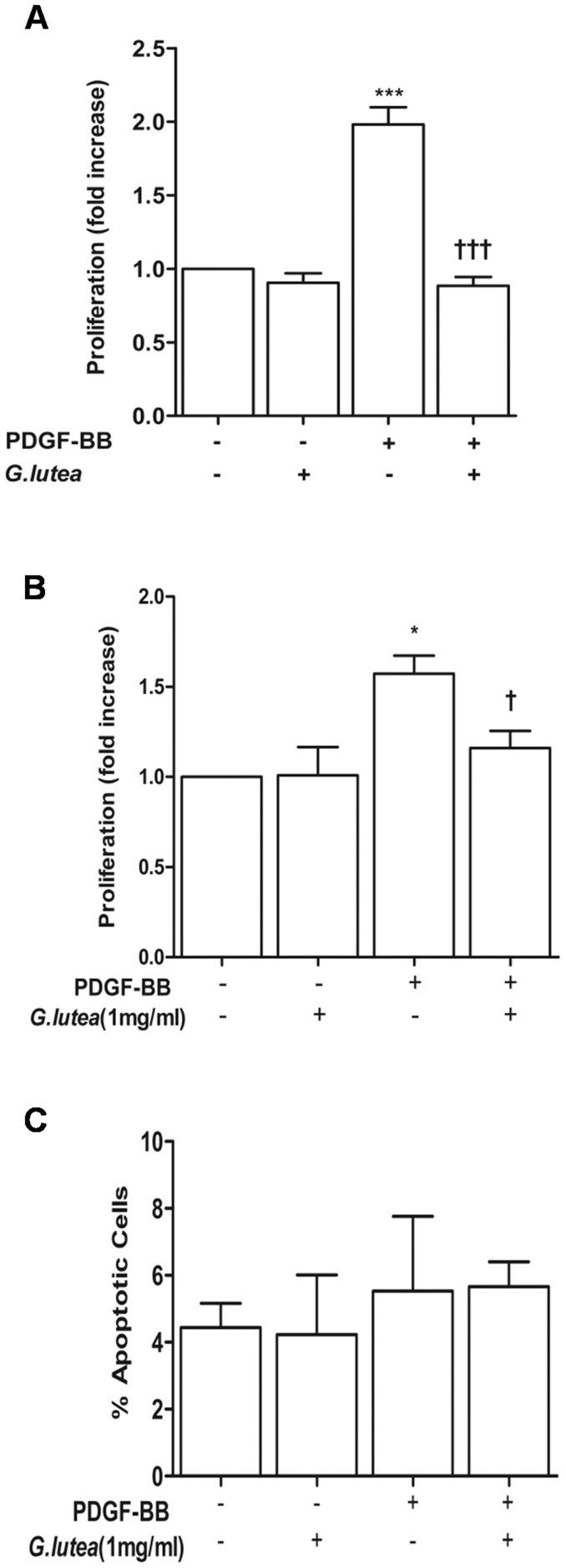
Effect of *G. lutea* extract (1 mg/ml) on proliferation and apoptosis. A) Effect of extract on PDGF-BB (20 ng/ml, 24 hours) induced proliferation of primary cultures of rat aortic smooth muscle cells (RASMCs), B) A7r5 and C) percentage of apoptotic RASMCs in response to experimental treatments. *P<0.05 and ***P<0.001 versus control and †P<0.05 and †††<0.001 versus PDGF treatment.

**Figure 3:**
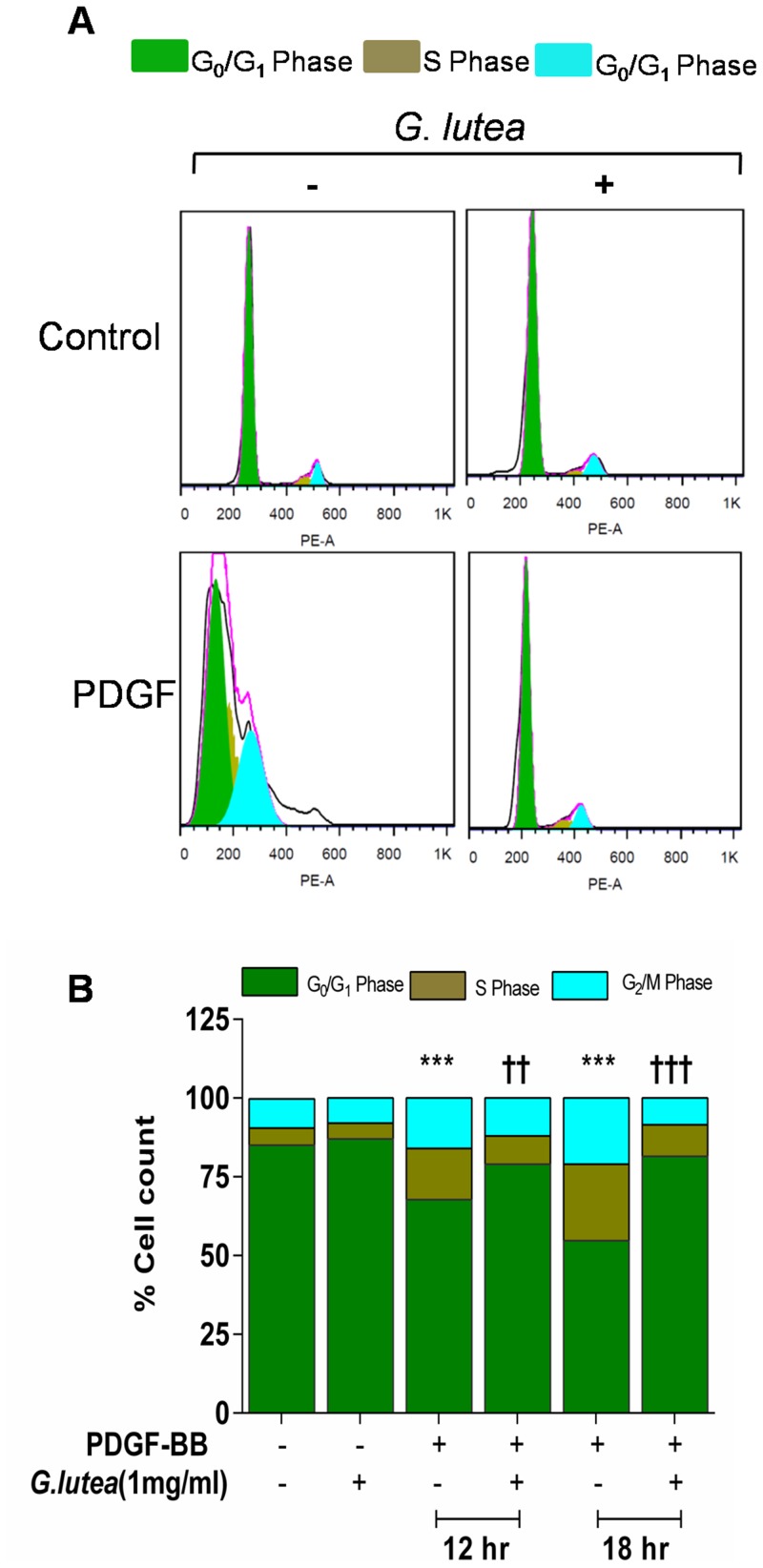
Cell cycle analysis of RASMCs. A) Representative flow cytogram depicting cells in various stages of cell cycle upon treatment with PDGF-BB (20 ng/ml) in presence and absence of *G. lutea* extract, and B) Bar graph summarizing data for four independent experiments. ***P<0.001 versus control, ††<0.01 and †††<0.001 versus PDGF treatment.

We then sought to determine whether co-incubation of smooth muscle cells with *G lutea* extract (1 mg/ml) for 24 hours blocks PDGF-BB (20 ng/ml) induced proliferation. As seen in figure 2, the extract significantly blocked PDGF-BB induced conversion of resazurin (alamar blue reagent) to its reduced form resorufin both in primary cultures of rat aortic smooth muscle cells (fig. 2A) and A7r5 cell line (fig. 2B). This effect was however not due to induction of apoptosis which was scored as Annexin V labeling (fig. 2C), neither was it due to interference of resazurin indicator with extract alone (data not shown). We then analyzed the effect of extract on cell cycle progression. Over-night serum starvation was done to achieve 85±2% synchronization of cells in G_0_/G_1_ phase. Cells were then treated with 20 ng/ml PDGF-BB and were analyzed at 12 and 18 hours post treatment for entry into S and G_2_/M phase using propidium iodide labeling (fig. 3A). The percentage of cells in S phase increased from 5.5±2% in control cells to 16.25±1.1% and 24.25±1.1% at 12 and 18 hours respectively in response to PDGF-BB (fig. 3B). While number of cells in G_2_/M phase increased from 9.25±0.75% at control to 16.00±1.3% at 12 hours and 21.00±1.6% at 18 hours. Treatment with *G lutea* extract significantly inhibited entry of synchronized cells in to S and G_2_/M phase in response to PDGF-BB as seen in figure 3A and B.

### 3.3 PDGF-BB Activates ERK1/2-nitric Oxide Axis

**Figure 4:**
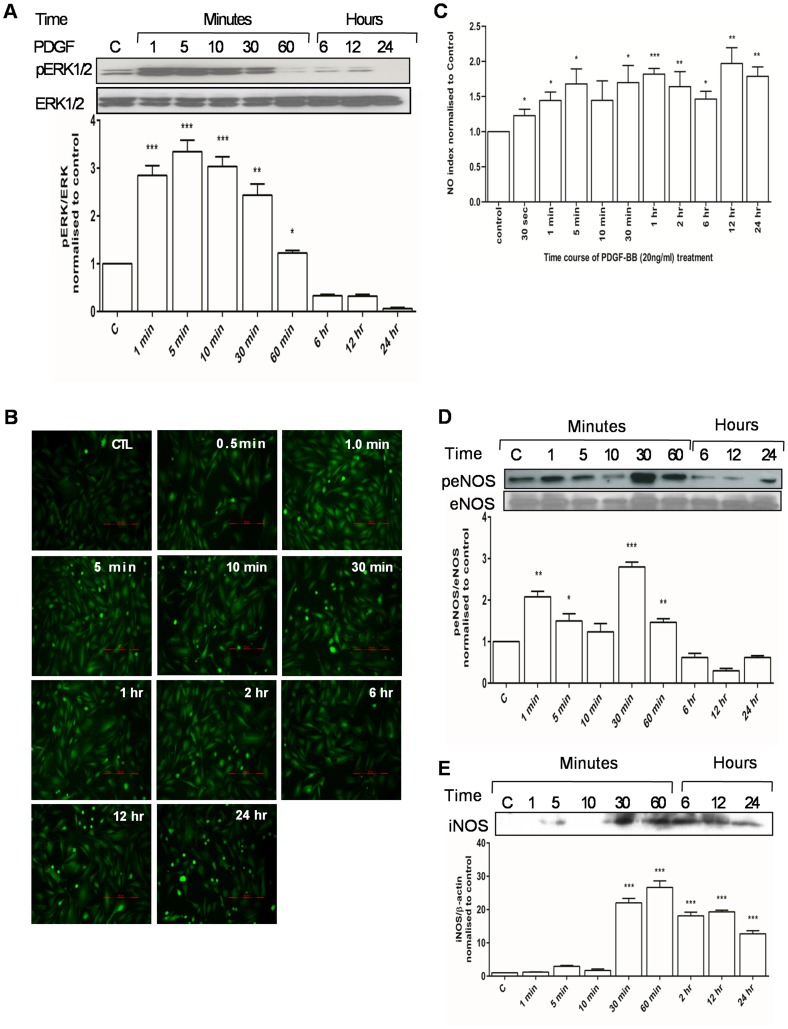
Effect of PDGF-BB (20 ng/ml) on ERK1/2 and NO signaling. A) Time course of ERK1/2 activation in response to PDGF, B&C) Time course of generation of intracellular nitric oxide in response to PDGF, D) Activation of eNOS through phosphorylation of Ser^1177^ residue in response to PDGF and E) PDGF-induced expression of iNOS. Bar graphs summarize data for a minimum of four independent experiments. *P<0.05, **P<0.01 and ***P<0.001 versus control.

**Figure 5:**
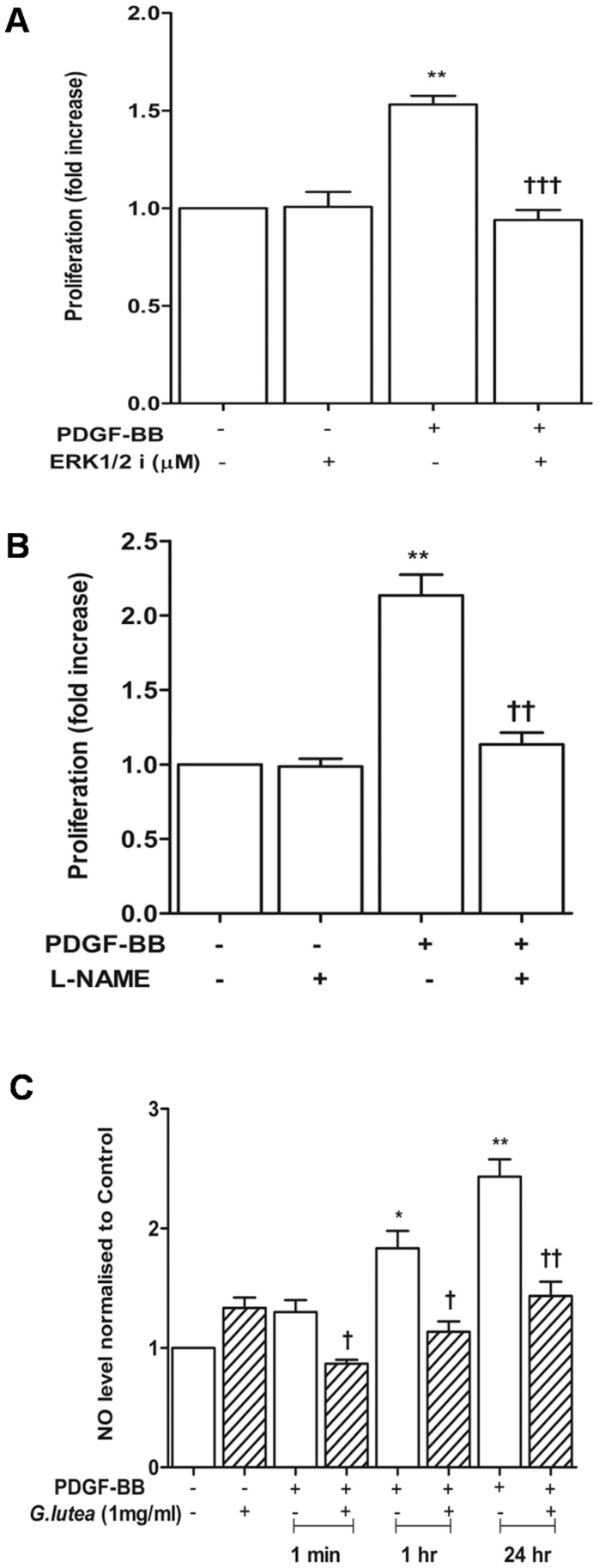
Role of ERK1/2-NO axis on PDGF-induced proliferation of RASMCs and effect of *G lutea* on PDGF induced NO index. A) Effect of ERK inhibitor 328000 (1 µmole/L) on PDGF induced cell proliferation, B) Effect of NOS inhibitor L-NAME (10 µmole/L) on PDGF induced cell proliferation, C) Effect of *G lutea* extract on generation of cellular NO in response to PDGF. *P<0.05 versus control and †<0.05, ††<0.01 and †††<0.001 versus corresponding PDGF treatment.

PDGF-BB treatment time dependently increased the activation of ERK1/2 which was measured as increase in Threonine^202^ and Tyrosine^204^ phosphorylation of ERK1 and dual phosphorylation of ERK2 at Threonine^185^ and Tyrosine^187^ (fig. 4A). ERK1/2 activation was seen from one minute onwards and was observed till 30 minutes. PDGF treatment also increased levels of intracellular nitric oxide (NO) as early as 30 seconds (fig. 4B&C). Early increase in nitric oxide coincided with Akt mediated Ser^1177^ phosphorylation of endothelial isoform of nitric oxide synthase (eNOS) (fig. 4D). This residue in eNOS activates the NO synthase activity [21]. An increase in the expression of iNOS was also seen in response to PDGF from 30 minute onwards (fig. 4E). Inhibition of both ERK1/2 and nitric oxide synthases via 328000 and L-NAME respectively blocked PDGF-BB induced proliferation (fig. 5A&B). We then determined the effect of root extract on PDGF-BB induced cellular NO. The *G lutea* root extract, completely blocked cellular NO induced by PDGF-BB (fig. 5C).

### 3.4 *G lutea* Extract Blocks PDGF-BB Induced Cell Signaling

**Figure 6:**
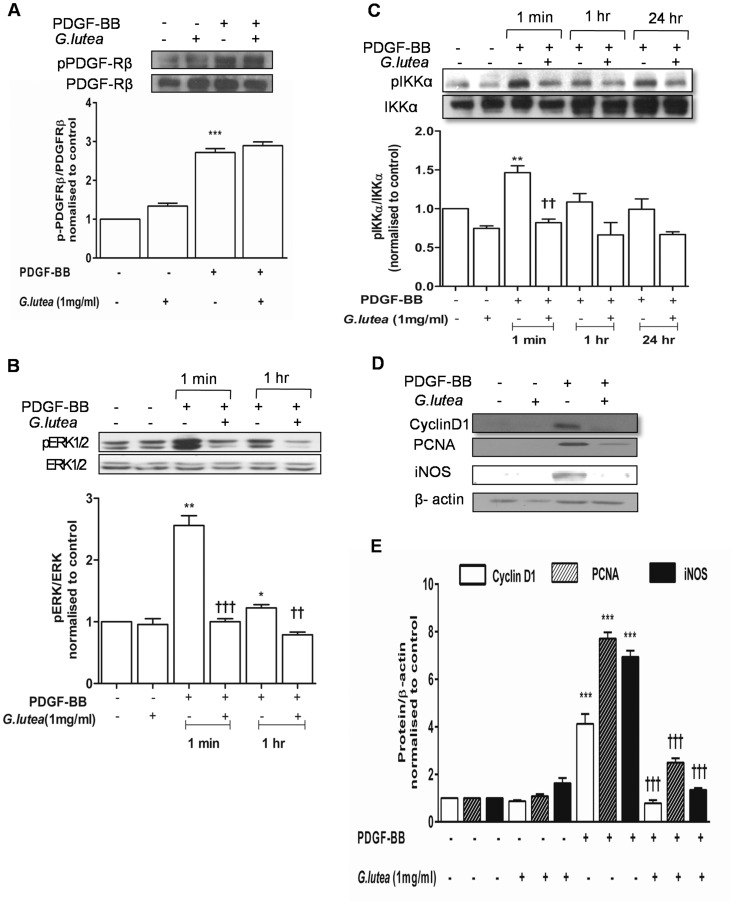
Effect of *G lutea* extract on PDGF-induced cell signaling in RASMCs. A) PDGFR-β phosphorylation, B) ERK1/2 activation, C) Phosphorylation of IKKα and D&E) Representative blot and bar graph indicating expression of cyclin D1, PCNA and iNOS for a minimum of three independent experiments. *<0.05, **P<0.01 and ***P<0.001 versus control and ††<0.01 and †††<0.001 versus PDGF treatment.

We then sought to determine the intracellular signaling affected by the root extract. The aqueous extract failed to block PDGF induced PDGF receptor tyrosine auto-phosphorylation at Tyr^751^ (fig. 6A) suggesting that its mode of action is down-stream of the receptor. However, it blocked PDGF-BB induced ERK1/2 activation (fig. 6B). Consequently the phosphorylation of IKKα a downstream target of ERK1/2 was also blocked by the extract (fig. 6C). Since IKK-NFκB axis is directly involved in activating transcription of iNOS [22], we determined the effect of the extract on PDGF-BB induced expression of iNOS. Indeed the extract blocked PDGF induced expression of iNOS (fig. 6D&E). Among the cell cycle regulators, the *G lutea* extract decreased PDGF-BB induced expression of PCNA and cyclin D1 (fig. 6D&E).

### 3.5 Docking Analysis with Extract Components

**Figure 7:**
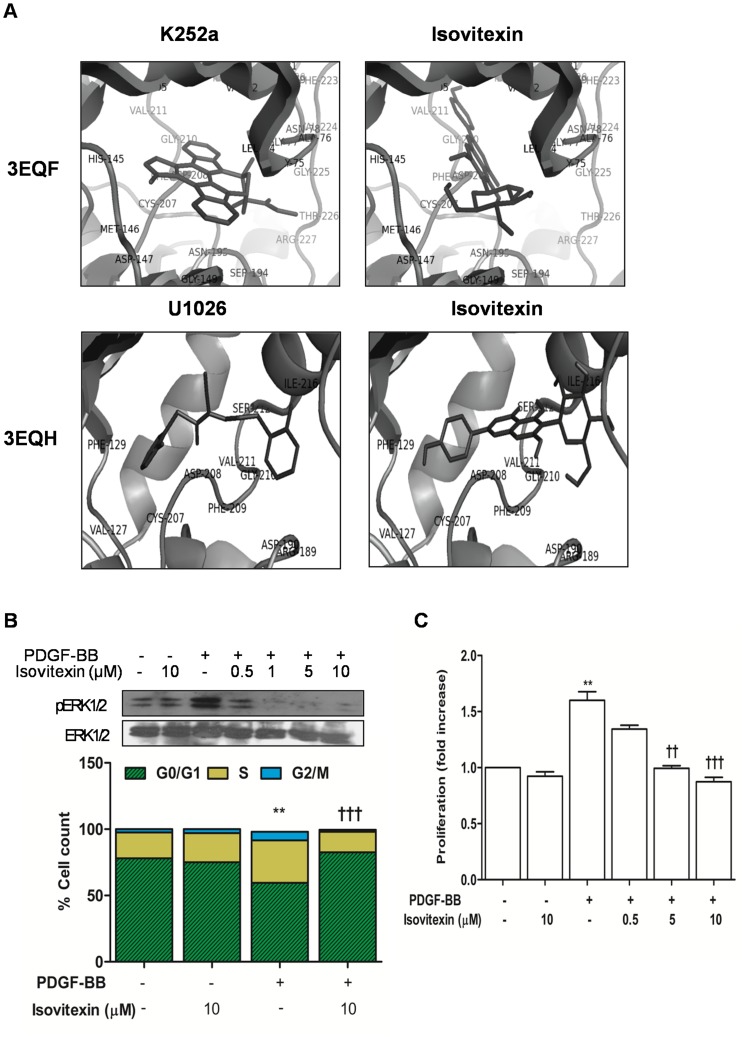
Effect of isovitexin on PDGF- induced RASMC proliferation. A) Docking analysis revealing binding pocket of isovitexin on MEK1, B) Effect of isovitexin (0.5–10 µmol/L) on PDGF induced ERK1/2 activation and cell cycle progress and C) Effect of isovitexin on PDGF induced cell proliferation measured through alamar blue assay. Bar graphs summarize data for a minimum of three independent experiments. **P<0.01 versus control and ††<0.01 and †††<0.001 versus PDGF treatment.

Since MEK1 is the known dual specificity kinase which phosphorylates the threonine and tyrosine residues in the activation loopes of ERK1 and ERK2 in order to activate them [23], [24], we set out to determine if any of the extract components interact with MEK1 through docking analysis. Due to lack of crystal structure for MEK2 and ERK1 we could not perform the analysis on these molecules. Using the Lamarckian algorithm of the AutoDock4 program blind and rigid docking of the extract constituents were done with x-ray structure of human mitogen-activated protein kinase kinase1 (MEK1) as described in methods. It should be noted that human MEK1 shares considerable homology with rat MEK1. Figure 7A depicts complexes of known inhibitors K252a and U1026 as well as isovitexin with MEK1. Among the components tested the best binding energies were predicted for isovitexin as seen in table 1. Isovitexin was found to bind to similar sites as K252a (for PDB id: 3EQF) and U1026 (PDB id: 3EQH) in MEK1 (fig. 7A). Residues which were found to be in close contact with isovitexin in 3EQF were Lys^97^, Leu^115^, Leu^118^, Val^127^, Met^143^, Glu^144^, His^145^, Met^146^, Leu^197^ Cys^207^, Asp^208^, Phe^209^, Gly^210^ and Val^211^. Among these, isovitexin exhibits propensity of hydrogen bonding with Lys^97^, Met^146^, Asp^208^ and Val^211^. Residues which were in close contact with isovitexin in 3EQH were Lys^97^, Leu^118^, Ile^141^, Asp^208^, Phe^209^, Val^211^, Leu^215^, Ile^216^, Met^219^ and Ala^220^. Of these, isovitexin exhibits possibility of hydrogen bonding with Val^211^. We then set out to determine experimentally whether purified isovitexin indeed blocks PDGF-BB induced signaling and proliferation in primary cultures of RASMCs. As seen in figure 7B, isovitexin at varying concentrations successfully blocked PDGF-induced ERK1/2 activation and entry of cells in to S-Phase. Effect of isovitexin on PDGF-induced proliferation was also confirmed via alamar blue assay (fig. 7C).

**Table 1:** Predicted binding energies for *G lutea* constituents and known MEK1. inhibitors.

Ligand	Best predicted binding energy in Kcal/mol for 3EQF	Best predicted binding energy in Kcal/mol for 3EQH
Amarogentin	−6.39	−1.83
Gentiopicroside	−4.18	−4.02
Gentisin	−5.76	−5.89
**Isovitexin**	−**8.16**	−**7.38**
Swertiamarin	−3.8	−2.94
**K252A**	−**10.41**	−
**U1026**	−**8.85**	−**10.79**

## Discussion

**Figure 8:**
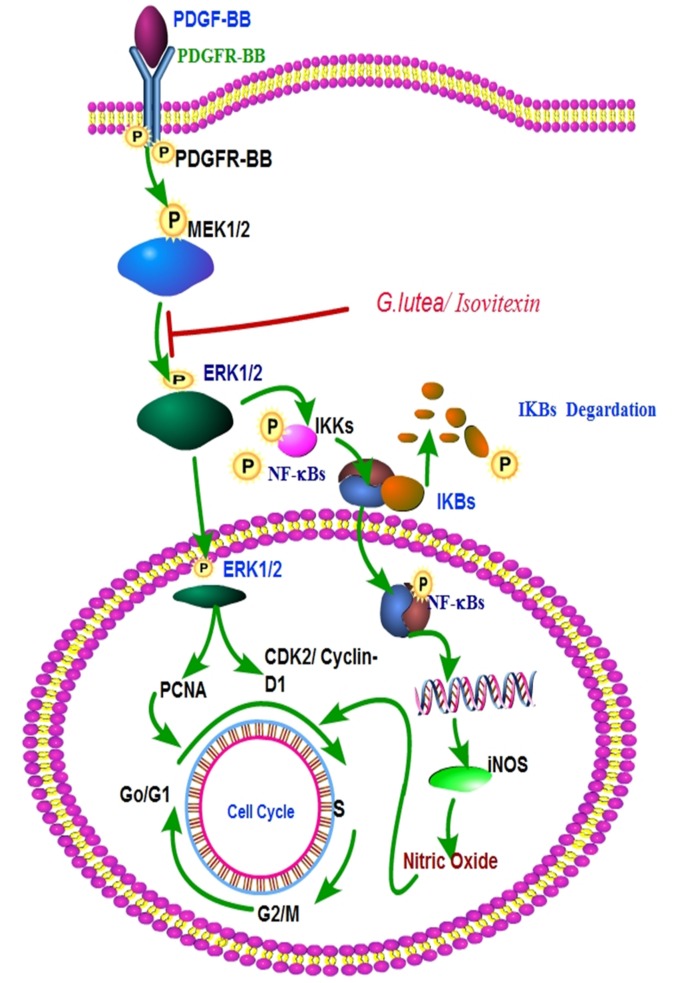
Summary figure depicting the pathway blocked by *G lutea* extract to prevent PDGF-induced RASMC proliferation.

Epidemiological studies demonstrate an inverse correlation between intake of dietary polyphenols and progression of chronic proliferative diseases such as cancer and atherosclerosis [25]. Proliferation and migration of VSMCs in response to PDGF triggers intimal thickening post-angioplasty [2]. Use of PDGF receptor antagonists blocks both in vitro proliferation of VSMCs and atherosclerosis in animal models [2], [26]. In the present study, we demonstrate that aqueous extract of *G lutea* roots effectively blocks PDGF-BB induced cell cycle progression by interfering with ERK1/2-iNOS signaling (as summarized in fig. 8). These effects of the extract were neither due to cytotoxicity nor due to apoptosis.

Entry and progression of cells through different stages of cell cycle is a tightly orchestrated process. It involves sequential activation and expression of regulatory proteins such as cyclins, cyclin dependent kinases (CDKs), CDK inhibitors, p53 and pRb [27], [28]. Activation of CDK2 and CDK4 in complex with cyclin D1 mediates progression of cells from G_0_/G_1_ to S phase. CDK2 in turn is involved in phosphorylation of pRb and accumulation of proliferating cell nuclear antigen (PCNA) [29]. Phosphorylation of pRb is also mediated by ERK1/2, wherein activated ERK1/2 directly phosphorylates pRb at Ser^780^ and Ser^795^ residues [30]. This event precedes cyclin D1 expression and is necessary for the release of transcription factors in order to promote DNA synthesis. We observed an increase in ERK1/2 phosphorylation upon PDGF-BB treatment, which was blocked by the *G lutea* extract. Consequently, the extract also blocked PDGF-BB induced expression of cyclin D1 and PCNA. However, the extract failed to block PDGF-BB induced PDGF-receptor tyrosine phosphorylation, thereby indicating that the site of action for the extract and its constituents is downstream of the receptor. Effects of ERK1/2 activation on cell cycle vary depending upon the type of cells involved. For instance, in fibroblasts ERK1/2 activation leads to G_1_ to S phase entry via cyclin D1 expression, while in epithelial cells it is required for G_2_ to M phase entry through cyclinB1 and c-fos [31]. Although we did not look for effect of *G lutea* extract on cyclinB1 and c-fos expression, given that the extract effectively blocks ERK1/2 activation we believe, it utilizes the same mechanism for blocking G_2_ to M phase entry of cells in response to PDGF-BB.

In the present study we also observed that PDGF-BB induced production of intracellular NO was significantly blocked by the *G lutea* extract. The exact effect of nitric oxide on cell cycle depends upon its local concentration. At low concentrations it promotes cell proliferation while at higher doses it induces cytostatic and/or apoptotic effects [32], [33]. Since in this study NOS inhibitor L-NAME blocked PDGF-BB induced cell proliferation we believe that intracellular NO in response to PDGF-BB promotes smooth muscle cell proliferation. Although the molecular mechanisms by which NO promotes cell proliferation are not well understood, recent studies have highlighted its ability to inhibit apoptosis by preventing the DNA binding activity of p53 [34] and through S-nitrosylation of caspases [35], [36].

The major sources of NO in cells are the various isoforms of NO synthases (NOS) such as iNOS (inducible isoform), nNOS (neuronal isoform) and eNOS (endothelial isoform) [37], [38]. Among these eNOS and nNOS are constitutively expressed while iNOS expression is transcriptionally induced in a MAPK-dependent manner upon agonist activation [37]. ERK1/2 activates the IKK-NFκB axis in order to activate transcription of iNOS [22]. We observed that PDGF-BB induced activation of ERK1/2 in vascular smooth muscle cells was responsible for increased expression of iNOS. Intriguingly, abundant expression and activation of ERK1/2 is seen in atheroma samples derived from hyperlipidemic mice [39]. Similarly, increased iNOS expression and activity is reported in VSMCs obtained from diabetic rats [40]. Involvement of iNOS in pathology of atherosclerosis is also supported by the fact that its deficiency reduces atherosclerotic plaques [41] and neo-intimal thickening [42] in rodent models. These reductions are either due to decreased LDL oxidation [41] or due to G_0_/G_1_ arrest of iNOS deficient VSMCs [43]. We also observed G_0_/G_1_ arrest of VSMCs in presence of *G lutea* extract. Additionally the extract blocked PDGF-BB induced iNOS expression. Given these observations, it is tempting to speculate that the active principles of *Gentiana lutea* root extract will prevent neo-intimal thickening by blocking ERK1/2-iNOS activation.

MEK1/2 is the only known upstream kinase which can activate ERK1/2 [23]. We hence carried out docking analysis of identified constituents of the *G lutea* extract with the available crystal structures of human MEK1. Two crystal structures of MEK1 as ternary complexes with known competitive inhibitor K252a (PDBID: 3EQF) and allosteric inhibitor U1026 (PDBID: 3EQH) were used for the analysis [20]. It was observed that among the individual components, isovitexin was seen to dock to the same binding pocket in MEK1 as K252a and U1026. It should be noted that inhibitor binding site in MEK1 is adjacent to the Mg^2+^ATP binding site and these two sites are physically separated by the side chains of Lys^97^ and Met^143^, while the other neighboring amino acids Leu^118^, Ileu^141^, and Phe^209^ form a deep hydrophobic pocket within the core kinase domain extending from amino acid 53 to 369 [20], [44]. Binding of K252a induces a conformational change due to hydrogen bonding with Glu^144^ and Met^146^ and thus affects catalytic activity of MEK1 [20]. On the other hand, although the exact molecular mechanism of allosteric inhibition of MEK1 is still unclear, crystal structure studies have predicted that binding of allosteric inhibitors adjacent to active site induces and stabilizes a basal and naturally occurring inactive form of MEK1 [44]. Conformational change induced by these allosteric inhibitors breaks a conserved ion pair formation between Glu^114^ and Lys^97^ in the ATP binding site of the enzyme. Among the *G lutea* components analyzed, isovitexin was found to dock to the same site as K252a and U1026 and demonstrated a propensity of hydrogen bonding with catalytic residues Lys^97^, Met^146^, Asp^208^, or Val^211^. Furthermore in cell culture experiment isovitexin successfully blocked PDGF induced proliferation of RASMCs.

Plant derived polyphenols and carotenoids delay the onset and progression of atherosclerosis by modulating serum lipid levels and by reducing oxidative stress and lipid peroxidation. Some of these also block VSMC proliferation such as ellagic acid [45], Naringenin [46], luteolin [47] and lutein [48]. Binding of PDGF-BB to its cognate receptor followed by auto-phosphorylation, triggers generation of intracellular reactive oxygen species (ROS) [49]. ROS then reversibly inactivate protein tyrosine phosphatases (PTPase), the known negative regulators of PDGF receptor signaling [50]. Alternatively these free radicals activate ERK1/2 pathway by activating ERK1/2 phosphorylating kinases such as Src, Pyk2 and Syk [51]. Since polyphenolic content of an extract imparts greater antioxidant capacity and given that both water and ethanol extracts of *Gentaian lutea* roots have high polyphenol content as already reported by us [15], it is likely that the extract through its radical scavenging activity is directly modulating PDGF induced ERK1/2 activation. Additionally others have shown that polyphenols obtained from numerous other Gentiana species act as antioxidants and effectively block lipid peroxidation [52]–[55]. As the receptor tyrosine phosphorylation was unaltered by the extract (fig. 5A), we believe that the anti-oxidant effect of the extract would lie downstream of the receptor but upstream of ERK1/2 activation. Thus antioxidants such as isovitexin [56] and gentisin present in the water extract may inactivate these kinases thereby blocking ERK1/2 activation. This however needs to be tested in our future studies.

Diabetes increases the risk of atherosclerosis. Hyperglycemia observed in these patients favors glucose metabolism through polyol pathway and induces formation of advanced glycation end products (AGEs) [57]. These pathways along with pro-inflammatory cytokines and growth factors promote diabetic micro- and macro-vascular complications. Aldose reductase (AR) the rate limiting enzyme in ployol pathway, upon over-expression, accelerates atherosclerotic lesions in apoE−/− mice [58]. Furthermore, its inhibition successfully blocks bFGF, angiotensin II, AGEs, hyperglycemia and PDGF-AB induced proliferation of vascular smooth muscle cells [59]–[61]. These effects of AR inhibitors are through their ability to attenuate ERK1/2-NFκB signaling leading to cell cycle arrest at G1 phase [61]. Intriguingly, the methanol and ether extracts of *Gentiana lutea* roots were recently shown by our co-authors to inhibit rat and human isoforms of AR, thereby preventing sorbitol accumulation under high glucose conditions [14]. Molecular docking studies identified amarogentin as a potential inhibitor of AR [14]. The present study identifies isovitexin as yet another anti-atherosclerotic agent in aqueous extract of *Gentiana lutea* roots. Similar studies on cancer cell lines have demonstrated the anti-tumor activity of root extracts of *Gentiana triflora* [62], but to the best of our knowledge this is the first study demonstrating anti-proliferative effects of *Gentiana lutea* extract on vascular smooth muscle cells. Although nothing is reported regarding the anti-diabetic effects of *Gentiana lutea*, isoorientin obtained from another *Gentianaceae* member, *Gentiana oliveri*, exhibits hypoglycemic and anti-hyperlipidemic effects in streptozotocin induced diabetic rats [63]. Thus it becomes imperative to study even the hypoglycemic effects of *Gentiana lutea* extracts in future. Recent work by Nastasijević et.al., has shown that gentiopicroside, isovitexin and amarogentin present in the root extracts of *Gentiana lutea* are effective inhibitors of myeloperoxidase [64]. It should be noted that myeloperoxidase is an early marker of vascular dysfunction and plays a crucial role in oxidative modification of LDL and hence in pathogenesis of atherosclerosis [65]. Given these observations and our current study we propose that Gentiana extracts and their constituents can provide drug leads to come up with effective anti-atherosclerosis therapy in future.

## Conclusion

In conclusion, aqueous root extract of *Gentiana lutea* and its constituent isovitexin effectively blocks PDGF-BB induced proliferation of rat aortic smooth muscle cells by blocking ERK1/2 activation and consequent iNOS expression.

## References

[1] Waltenberger J (1997) Modulation of growth factor action: implications for the treatment ofcardiovascular diseases. Circulation 96: 4083–4094. 9403634 10.1161/01.cir.96.11.4083

[2] Andrae J, Gallini R, Betsholtz C (2008) Role of platelet-derived growth factors in physiology and medicine. Genes Dev 22: 1276–1312 22/10/1276 [pii];10.1101/gad.1653708 [doi]. 18483217 10.1101/gad.1653708 PMC2732412

[3] Claesson-Welsh L (1994) Platelet-derived growth factor receptor signals. J Biol Chem 269: 32023–32026. 7798193

[4] Scalbert A, Manach C, Morand C, Remesy C, Jimenez L (2005) Dietary polyphenols and the prevention of diseases. Crit Rev Food Sci Nutr 45: 287–306 10.1080/1040869059096 [doi]. 16047496 10.1080/1040869059096

[5] Brahmachari G, Mondal S, Gangopadhyay A, Gorai D, Mukhopadhyay B, et al (2004) Swertia (Gentianaceae): chemical and pharmacological aspects. Chem Biodivers 1: 1627–1651 10.1002/cbdv.200490123 [doi]. 17191805 10.1002/cbdv.200490123

[6] Nadinica EGSRABCDSAUC (1999) Topical anti-inflammatory activity of Gentianella achalensis. 70: 166–171.

[7] Jankovic T, Savikin K, Menkovic N, Aljancic I, Leskovac A, et al (2008) Radioprotective effects of Gentianella austriaca fractions and polyphenolic constituents in human lymphocytes. Planta Med 74: 736–740 10.1055/s-2008-1074524 [doi]. 18446672 10.1055/s-2008-1074524

[8] Leskovac A, Joksic G, Jankovic T, Savikin K, Menkovic N (2007) Radioprotective properties of the phytochemically characterized extracts of Crataegus monogyna, Cornus mas and Gentianella austriaca on human lymphocytes in vitro. Planta Med 73: 1169–1175 10.1055/s-2007-981586 [doi]. 17764067 10.1055/s-2007-981586

[9] Menkovic N, Juranic Z, Stanojkovic T, Raonic-Stevanovic T, Savikin K, et al (2010) Radioprotective activity of Gentiana lutea extract and mangiferin. Phytother Res 24: 1693–1696 10.1002/ptr.3225 [doi]. 21031630 10.1002/ptr.3225

[10] Aberham A, Schwaiger S, Stuppner H, Ganzera M (2007) Quantitative analysis of iridoids, secoiridoids, xanthones and xanthone glycosides in Gentiana lutea L. roots by RP-HPLC and LC-MS. J Pharm Biomed Anal 45: 437–442 S0731–7085(07)00391-3 [pii];10.1016/j.jpba.2007.07.001 [doi]. 17697760 10.1016/j.jpba.2007.07.001

[11] Toriumi Y, Kakuda R, Kikuchi M, Yaoita Y, Kikuchi M (2003) New triterpenoids from Gentiana lutea. Chem Pharm Bull (Tokyo) 51: 89–91. 12520135 10.1248/cpb.51.89

[12] Schmieder A, Schwaiger S, Csordas A, Backovic A, Messner B, et al (2007) Isogentisin–a novel compound for the prevention of smoking-caused endothelial injury. Atherosclerosis 194: 317–325 S0021–9150(06)00650-2 [pii];10.1016/j.atherosclerosis.2006.10.019 [doi]. 17141243 10.1016/j.atherosclerosis.2006.10.019

[13] Rojas A, Bah M, Rojas JI, Gutierrez DM (2000) Smooth muscle relaxing activity of gentiopicroside isolated from Gentiana spathacea. Planta Med 66: 765–767 10.1055/s-2000-9774 [doi]. 11199140 10.1055/s-2000-9774

[14] Akileshwari C, Muthenna P, Nastasijevic B, Joksic G, Petrash JM, et al (2012) Inhibition of aldose reductase by Gentiana lutea extracts. Exp Diabetes Res 2012: 147965 10.1155/2012/147965 [doi]. 22844269 10.1155/2012/147965 PMC3403369

[15] Nastasijevic B, Lazarevic-Pasti T, Dimitrijevic-Brankovic S, Pasti I, Vujacic A, et al (2012) Inhibition of myeloperoxidase and antioxidative activity of Gentiana lutea extracts. J Pharm Biomed Anal 66: 191–196 S0731–7085(12)00191–4 [pii];10.1016/j.jpba.2012.03.052 [doi]. 22521634 10.1016/j.jpba.2012.03.052

[16] Brown C, Pan X, Hassid A (1999) Nitric oxide and C-type atrial natriuretic peptide stimulate primary aortic smooth muscle cell migration via a cGMP-dependent mechanism: relationship to microfilament dissociation and altered cell morphology. Circ Res 84: 655–667. 10189353 10.1161/01.res.84.6.655

[17] Nickel T, Deutschmann A, Hanssen H, Summo C, Wilbert-Lampen U (2009) Modification of endothelial biology by acute and chronic stress hormones. Microvasc Res 78: 364–369 S0026–2862(09)00220–9 [pii];10.1016/j.mvr.2009.07.008 [doi]. 19664643 10.1016/j.mvr.2009.07.008

[18] Ahmed SA, Gogal RM Jr, Walsh JE (1994) A new rapid and simple non-radioactive assay to monitor and determine the proliferation of lymphocytes: an alternative to [3H]thymidine incorporation assay. J Immunol Methods 170: 211–224. 8157999 10.1016/0022-1759(94)90396-4

[19] Morris GM, Huey R, Lindstrom W, Sanner MF, Belew RK, et al (2009) AutoDock4 and AutoDockTools4: Automated docking with selective receptor flexibility. J Comput Chem 30: 2785–2791 10.1002/jcc.21256 [doi]. 19399780 10.1002/jcc.21256 PMC2760638

[20] Fischmann TO, Smith CK, Mayhood TW, Myers JE, Reichert P, et al (2009) Crystal structures of MEK1 binary and ternary complexes with nucleotides and inhibitors. Biochemistry 48: 2661–2674 10.1021/bi801898e [doi];10.1021/bi801898e [pii]. 19161339 10.1021/bi801898e

[21] Dimmeler S, Fleming I, Fisslthaler B, Hermann C, Busse R, et al (1999) Activation of nitric oxide synthase in endothelial cells by Akt-dependent phosphorylation. Nature 399: 601–605 10.1038/21224 [doi]. 10376603 10.1038/21224

[22] Jiang B, Brecher P, Cohen RA (2001) Persistent activation of nuclear factor-kappaB by interleukin-1beta and subsequent inducible NO synthase expression requires extracellular signal-regulated kinase. Arterioscler Thromb Vasc Biol 21: 1915–1920. 11742864 10.1161/hq1201.099424

[23] Shaul YD, Gibor G, Plotnikov A, Seger R (2009) Specific phosphorylation and activation of ERK1c by MEK1b: a unique route in the ERK cascade. Genes Dev 23: 1779–1790 23/15/1779 [pii];10.1101/gad.523909 [doi]. 19651986 10.1101/gad.523909 PMC2720265

[24] Wortzel I, Seger R (2011) The ERK Cascade: Distinct Functions within Various Subcellular Organelles. Genes Cancer 2: 195–209 10.1177/1947601911407328 [doi];10.1177_1947601911407328 [pii]. 21779493 10.1177/1947601911407328 PMC3128630

[25] Arts IC, Hollman PC (2005) Polyphenols and disease risk in epidemiologic studies. Am J Clin Nutr 81: 317S–325S. 81/1/317S [pii]. 10.1093/ajcn/81.1.317S 15640497

[26] Myllarniemi M, Frosen J, Calderon Ramirez LG, Buchdunger E, Lemstrom K, et al (1999) Selective tyrosine kinase inhibitor for the platelet-derived growth factor receptor in vitro inhibits smooth muscle cell proliferation after reinjury of arterial intima in vivo. Cardiovasc Drugs Ther 13: 159–168. 10372232 10.1023/a:1007700629728

[27] Sanchez I, Dynlacht BD (2005) New insights into cyclins, CDKs, and cell cycle control. Semin Cell Dev Biol 16: 311–321 S1084–9521(05)00031–5 [pii];10.1016/j.semcdb.2005.02.007 [doi]. 15840440 10.1016/j.semcdb.2005.02.007

[28] Golias CH, Charalabopoulos A, Charalabopoulos K (2004) Cell proliferation and cell cycle control: a mini review. Int J Clin Pract 58: 1134–1141. 15646411 10.1111/j.1742-1241.2004.00284.x

[29] Akiyama T, Ohuchi T, Sumida S, Matsumoto K, Toyoshima K (1992) Phosphorylation of the retinoblastoma protein by cdk2. Proc Natl Acad Sci U S A 89: 7900–7904. 1518810 10.1073/pnas.89.17.7900 PMC49822

[30] Guo J, Sheng G, Warner BW (2005) Epidermal growth factor-induced rapid retinoblastoma phosphorylation at Ser780 and Ser795 is mediated by ERK1/2 in small intestine epithelial cells. J Biol Chem 280: 35992–35998 M504583200 [pii];10.1074/jbc.M504583200 [doi]. 16126730 10.1074/jbc.M504583200

[31] Dumesic PA, Scholl FA, Barragan DI, Khavari PA (2009) Erk1/2 MAP kinases are required for epidermal G2/M progression. J Cell Biol 185: 409–422 jcb.200804038 [pii];10.1083/jcb.200804038 [doi]. 19414607 10.1083/jcb.200804038 PMC2700391

[32] Choi BM, Pae HO, Jang SI, Kim YM, Chung HT (2002) Nitric oxide as a pro-apoptotic as well as anti-apoptotic modulator. J Biochem Mol Biol 35: 116–126. 16248976 10.5483/bmbrep.2002.35.1.116

[33] Villalobo A (2006) Nitric oxide and cell proliferation. FEBS J 273: 2329–2344 EJB5250 [pii];10.1111/j.1742–4658.2006.05250.x [doi]. 16704409 10.1111/j.1742-4658.2006.05250.x

[34] Calmels S, Hainaut P, Ohshima H (1997) Nitric oxide induces conformational and functional modifications of wild-type p53 tumor suppressor protein. Cancer Res 57: 3365–3369. 9269997

[35] Jiang ZL, Fletcher NM, Diamond MP, Abu-Soud HM, Saed GM (2009) S-nitrosylation of caspase-3 is the mechanism by which adhesion fibroblasts manifest lower apoptosis. Wound Repair Regen 17: 224–229 WRR459 [pii];10.1111/j.1524–475X.2009.00459.x [doi]. 19320891 10.1111/j.1524-475X.2009.00459.x PMC4529118

[36] Maejima Y, Adachi S, Morikawa K, Ito H, Isobe M (2005) Nitric oxide inhibits myocardial apoptosis by preventing caspase-3 activity via S-nitrosylation. J Mol Cell Cardiol 38: 163–174 S0022-2828(04)00315-3 [pii];10.1016/j.yjmcc.2004.10.012 [doi]. 15623433 10.1016/j.yjmcc.2004.10.012

[37] Geller DA, Billiar TR (1998) Molecular biology of nitric oxide synthases. Cancer Metastasis Rev 17: 7–23. 9544420 10.1023/a:1005940202801

[38] Papapetropoulos A, Rudic RD, Sessa WC (1999) Molecular control of nitric oxide synthases in the cardiovascular system. Cardiovasc Res 43: 509–520. S0008–6363(99)00161–3 [pii]. 10.1016/s0008-6363(99)00161-3 10690323

[39] Hu Y, Dietrich H, Metzler B, Wick G, Xu Q (2000) Hyperexpression and activation of extracellular signal-regulated kinases (ERK1/2) in atherosclerotic lesions of cholesterol-fed rabbits. Arterioscler Thromb Vasc Biol 20: 18–26. 10634796 10.1161/01.atv.20.1.18

[40] Di PN, Di TP, Di SS, Giardinelli A, Pipino C, et al (2013) Increased iNOS activity in vascular smooth muscle cells from diabetic rats: Potential role of Ca(2+)/calmodulin-dependent protein kinase II delta 2 (CaMKIIdelta(2)). Atherosclerosis 226: 88–94 S0021–9150(12)00748–4 [pii];10.1016/j.atherosclerosis.2012.10.062 [doi]. 23177014 10.1016/j.atherosclerosis.2012.10.062

[41] Miyoshi T, Li Y, Shih DM, Wang X, Laubach VE, et al (2006) Deficiency of inducible NO synthase reduces advanced but not early atherosclerosis in apolipoprotein E-deficient mice. Life Sci 79: 525–531 S0024–3205(06)00098–1 [pii];10.1016/j.lfs.2006.01.043 [doi]. 16516241 10.1016/j.lfs.2006.01.043

[42] Chyu KY, Dimayuga P, Zhu J, Nilsson J, Kaul S, et al (1999) Decreased neointimal thickening after arterial wall injury in inducible nitric oxide synthase knockout mice. Circ Res 85: 1192–1198. 10590247 10.1161/01.res.85.12.1192

[43] Chyu KY, Dimayuga PC, Zhao X, Nilsson J, Shah PK, et al (2004) Altered AP-1/Ref-1 redox pathway and reduced proliferative response in iNOS-deficient vascular smooth muscle cells. Vasc Med 9: 177–183. 15675181 10.1191/1358863x04vm545oa

[44] Ohren JF, Chen H, Pavlovsky A, Whitehead C, Zhang E, et al (2004) Structures of human MAP kinase kinase 1 (MEK1) and MEK2 describe novel noncompetitive kinase inhibition. Nat Struct Mol Biol 11: 1192–1197 nsmb859 [pii];10.1038/nsmb859 [doi]. 15543157 10.1038/nsmb859

[45] Chang WC, Yu YM, Chiang SY, Tseng CY (2008) Ellagic acid suppresses oxidised low-density lipoprotein-induced aortic smooth muscle cell proliferation: studies on the activation of extracellular signal-regulated kinase 1/2 and proliferating cell nuclear antigen expression. Br J Nutr 99: 709–714 S0007114507831734 [pii];10.1017/S0007114507831734 [doi]. 18184451 10.1017/S0007114507831734

[46] Chen S, Ding Y, Tao W, Zhang W, Liang T, et al (2012) Naringenin inhibits TNF-alpha induced VSMC proliferation and migration via induction of HO-1. Food Chem Toxicol 50: 3025–3031 S0278–6915(12)00420–6 [pii];10.1016/j.fct.2012.06.006 [doi]. 22709785 10.1016/j.fct.2012.06.006

[47] Lang Y, Chen D, Li D, Zhu M, Xu T, et al (2012) Luteolin inhibited hydrogen peroxide-induced vascular smooth muscle cells proliferation and migration by suppressing the Src and Akt signalling pathways. J Pharm Pharmacol 64: 597–603 10.1111/j.2042–7158.2011.01438.x [doi]. 22420665 10.1111/j.2042-7158.2011.01438.x

[48] Lo HM, Tsai YJ, Du WY, Tsou CJ, Wu WB (2012) A naturally occurring carotenoid, lutein, reduces PDGF and H(2)O(2) signaling and compromised migration in cultured vascular smooth muscle cells. J Biomed Sci 19: 18 1423–0127–19–18 [pii];10.1186/1423–0127–19–18 [doi]. 22313606 10.1186/1423-0127-19-18 PMC3292940

[49] Kreuzer J, Viedt C, Brandes RP, Seeger F, Rosenkranz AS, et al (2003) Platelet-derived growth factor activates production of reactive oxygen species by NAD(P)H oxidase in smooth muscle cells through Gi1,2. FASEB J 17: 38–40 10.1096/fj.01–1036fje [doi];01–1036fje [pii]. 12424219 10.1096/fj.01-1036fje

[50] Kappert K, Sparwel J, Sandin A, Seiler A, Siebolts U, et al (2006) Antioxidants relieve phosphatase inhibition and reduce PDGF signaling in cultured VSMCs and in restenosis. Arterioscler Thromb Vasc Biol 26: 2644–2651 01.ATV.0000246777.30819.85 [pii];10.1161/01.ATV.0000246777.30819.85 [doi]. 16990553 10.1161/01.ATV.0000246777.30819.85

[51] Roskoski R Jr (2012) ERK1/2 MAP kinases: structure, function, and regulation. Pharmacol Res 66: 105–143 S1043–6618(12)00097–7 [pii];10.1016/j.phrs.2012.04.005 [doi]. 22569528 10.1016/j.phrs.2012.04.005

[52] Yang JL, Liu LL, Shi YP (2010) Phytochemicals and biological activities of Gentiana species. Nat Prod Commun 5: 649–664. 20433091

[53] Wu QX, Li Y, Shi YP (2006) Antioxidant phenolic glucosides from Gentiana piasezkii. J Asian Nat Prod Res 8: 391–396 W301786611Q72362 [pii];10.1080/10286020500172368 [doi]. 16864453 10.1080/10286020500172368

[54] Myagmar BE, Aniya Y (2000) Free radical scavenging action of medicinal herbs from Mongolia. Phytomedicine 7: 221–229. 11185733 10.1016/S0944-7113(00)80007-0

[55] Hudecova A, Kusznierewicz B, Hasplova K, Huk A, Magdolenova Z, et al.. (2012) Gentiana asclepiadea exerts antioxidant activity and enhances DNA repair of hydrogen peroxide- and silver nanoparticles-induced DNA damage. Food Chem Toxicol. S0278–6915(12)00431–0 [pii];10.1016/j.fct.2012.06.017 [doi]. 10.1016/j.fct.2012.06.017 22721983

[56] Cao D, Li H, Yi J, Zhang J, Che H, et al (2011) Antioxidant properties of the mung bean flavonoids on alleviating heat stress. PLoS One 6: e21071 10.1371/journal.pone.0021071 [doi];PONE-D-11-03702 [pii]. 21695166 10.1371/journal.pone.0021071 PMC3112222

[57] Baba SP, Barski OA, Ahmed Y, O'Toole TE, Conklin DJ, et al (2009) Reductive metabolism of AGE precursors: a metabolic route for preventing AGE accumulation in cardiovascular tissue. Diabetes 58: 2486–2497 db09–0375 [pii];10.2337/db09–0375 [doi]. 19651811 10.2337/db09-0375 PMC2768164

[58] Srivastava S, Vladykovskaya E, Barski OA, Spite M, Kaiserova K, et al (2009) Aldose reductase protects against early atherosclerotic lesion formation in apolipoprotein E-null mice. Circ Res 105: 793–802 CIRCRESAHA.109.200568 [pii];10.1161/CIRCRESAHA.109.200568 [doi]. 19729598 10.1161/CIRCRESAHA.109.200568 PMC3548455

[59] Dan Q, Wong R, Chung SK, Chung SS, Lam KS (2004) Interaction between the polyol pathway and non-enzymatic glycation on aortic smooth muscle cell migration and monocyte adhesion. Life Sci 76: 445–459 S0024–3205(04)00839–2 [pii];10.1016/j.lfs.2004.09.010 [doi]. 15530506 10.1016/j.lfs.2004.09.010

[60] Ramana KV, Chandra D, Srivastava S, Bhatnagar A, Aggarwal BB, et al (2002) Aldose reductase mediates mitogenic signaling in vascular smooth muscle cells. J Biol Chem 277: 32063–32070 10.1074/jbc.M202126200 [doi];M202126200 [pii]. 12063254 10.1074/jbc.M202126200

[61] Tammali R, Saxena A, Srivastava SK, Ramana KV (2010) Aldose reductase regulates vascular smooth muscle cell proliferation by modulating G1/S phase transition of cell cycle. Endocrinology 151: 2140–2150 en.2010–0160 [pii];10.1210/en.2010–0160 [doi]. 20308528 10.1210/en.2010-0160 PMC2869260

[62] Matsukawa K, Ogata M, Hikage T, Minami H, Shimotai Y, et al.. (2006) Antiproliferative activity of root extract from gentian plant (Gentiana triflora) on cultured and implanted tumor cells. Biosci Biotechnol Biochem 70: 1046–1048. JST.JSTAGE/bbb/70.1046 [pii]. 10.1271/bbb.70.1046 16636481

[63] Sezik E, Aslan M, Yesilada E, Ito S (2005) Hypoglycaemic activity of Gentiana olivieri and isolation of the active constituent through bioassay-directed fractionation techniques. Life Sci 76: 1223–1238 S0024–3205(04)00949-X [pii];10.1016/j.lfs.2004.07.024 [doi]. 15642593 10.1016/j.lfs.2004.07.024

[64] Nastasijevic B, Lazarevic-Pasti T, Dimitrijevic-Brankovic S, Pasti I, Vujacic A, et al (2012) Inhibition of myeloperoxidase and antioxidative activity of Gentiana lutea extracts. J Pharm Biomed Anal 66: 191–196 S0731–7085(12)00191–4 [pii];10.1016/j.jpba.2012.03.052 [doi]. 22521634 10.1016/j.jpba.2012.03.052

[65] Schindhelm RK, van der Zwan LP, Teerlink T, Scheffer PG (2009) Myeloperoxidase: a useful biomarker for cardiovascular disease risk stratification? Clin Chem 55: 1462–1470 clinchem.2009.126029 [pii];10.1373/clinchem.2009.126029 [doi]. 19556446 10.1373/clinchem.2009.126029

